# A New Deep Learning Method with Self-Supervised Learning for Delineation of the Electrocardiogram

**DOI:** 10.3390/e24121828

**Published:** 2022-12-15

**Authors:** Wenwen Wu, Yanqi Huang, Xiaomei Wu

**Affiliations:** 1 Center for Biomedical Engineering, School of Information Science and Technology, Fudan University, Shanghai 200433, China; 2 Academy for Engineering and Technology, Fudan University, Shanghai 200433, China; 3 Key Laboratory of Medical Imaging Computing and Computer Assisted Intervention (MICCAI) of Shanghai, Shanghai 200433, China; 4 Yiwu Research Institute of Fudan University, Yiwu 322000, China; 5 Shanghai Engineering Research Center of Assistive Devices, Shanghai 200433, China

**Keywords:** self-supervised learning, deep learning, electrocardiogram, ECG characteristic points

## Abstract

Heartbeat characteristic points are the main features of an electrocardiogram (ECG), which can provide important information for ECG-based cardiac diagnosis. In this manuscript, we propose a self-supervised deep learning framework with modified Densenet to detect ECG characteristic points, including the onset, peak and termination points of P-wave, QRS complex wave and T-wave. We extracted high-level features of ECG heartbeats from the QT Database (QTDB) and two other larger datasets, MIT-BIH Arrhythmia Database (MITDB) and MIT-BIH Normal Sinus Rhythm Database (NSRDB) with no human-annotated labels as pre-training. By applying different transformations to ECG signals, the task of discriminating signals before and after transformation was defined as the pretext task. Subsequently, the convolutional layer was frozen and the weights of the self-supervised network were transferred to the downstream task of characteristic point localizations on heart beats in the QT dataset. Finally, the mean ± standard deviation of the detection errors of our proposed self-supervised learning method in QTDB for detecting the onset, peak, and termination points of P-waves, the onset and termination points of QRS waves, and the peak and termination points of T-waves were −0.24 ± 10.04, −0.48 ± 11.69, −0.28 ± 10.19, −3.72 ± 8.18, −4.12 ± 13.54, −0.68 ± 20.42, and 1.34 ± 21.04. The results show that the deep learning network based on the self-supervised framework constructed in this manuscript can accurately detect the feature points of a heartbeat, laying the foundation for automatic extraction of key information related to ECG-based diagnosis.

## 1. Introduction

Electrocardiogram (ECG) is an important tool in the diagnosis of cardiovascular diseases. With the widespread use of various ECG detection devices in the clinic, a large amount of ECG data is generated. Combining clinical ECG with computer technology to accomplish automatic identification of arrhythmia types can effectively diagnose heart diseases and reduce mortality [1]. ECG reflects the course of electrical activity of heart excitement. Each heartbeat contains P waves, QRS complex waves and T waves. The ECG physician draws diagnostic conclusions by observing the width, amplitude, morphology, and interrelationship of each wave and line segment of the ECG. This method of analysis is based on identifying characteristic points such as the onset, peak and termination of each wave of the ECG signal [2]. However, the variety of arrhythmia types and the richness of ECG morphology in different patients often make the clinical assessment workload of the professional enormous and sometimes physicians has a tendency to be subjective. In recent years, as deep-learning-based neural network models have achieved great success in a variety of fields such as natural language processing, computer vision, and biomedical signal processing. Deep learning research works for ECG data have gradually become popular, and these works have achieved comparable or even better classification performance than traditional methods. The main advantage of deep learning methods over machine learning is that there is no need for manual feature extraction, and deep learning models are able to perform feature extraction automatically and implicitly based on large raw data sets.

However, this automatic analysis method based on deep learning usually gives discriminative results directly after inputting ECG signals. Despite the high discriminative accuracy of some studies, it is not in line with the idea of evidence-based medicine due to the lack of interpretability. For this reason, our experiments are based on enhancing the interpretability of deep learning to process ECG signal operations, aiming to identify characteristic points such as the onset and termination points of each wave, and the peak of the ECG signal.

Traditional methods for detecting ECG characteristic points include wavelet transform, hidden Markov model, adaptive filtering, etc. Li [3] detected the position of QRS waves based on wavelet transform by calculating the relationship between their wavelet modulus maximum pairs. Juan et al. [4] used wavelet transformation to detect the onset and termination points of complex waves of heartbeats, the peaks of single waves, and the onset and termination points of P and T waves one by one. Martinez [5] proposed an algorithm based on phase volume transformation to calculate the mean ± standard deviation of P-wave onset, peak and termination points, QRS-wave onset and termination points, T-wave peak and termination points on a public ECG database. Furthermore, the results were 2.6 ± 14.5 ms, 32 ± 25.7 ms, 0.7 ± 14.7 ms, −0.2 ± 7.2 ms, 2.5 ± 8.9 ms, 5.3 ± 12.9 ms, 5.8 ± 22.7 ms. Clavier et al. [6] used the Hidden Markov method to represent a beat of the P-wave. Then, a set of parameters was calculated from the P-wave to detect patients prone to atrial fibrillation(AF) with a sensitivity of approximately 65% to 70%. Traditional method may perform poorly on large datasets, and deep learning is a branch of machine learning that is often used for detection of medium or large datasets. Camps et al. [7] used a CNN-based approach to localize QRS onset and termination points with root mean square error (RMSE) of 12.1 ± 0.5 ms and 18.5 ± 1.1 ms, respectively. Guillermo et al. [8] used a U-Net network structure for localization of P-wave, QRS-wave and T-wave onset and termination points. By segmenting a heartbeat into different regions and then performing characteristic point localization, the mean ± standard deviation of detection errors were 1.54 ± 22.89 ms, 0.32 ± 4.01 ms, −0.07 ± 8.37 ms, 3.64 ± 12.55 ms, 21.57 ± 66.29 ms, and 4.55 ± 31.11 ms, respectively. Abrishami et al. [9] used LSTM-based segmentation method for P-wave, QRS-wave, T-wave and other waves with an accuracy of more than 90%.

However, fully supervised learning of deep learning requires a large amount of manually labeled data, which is very time-consuming and labor-intensive. At the same time, training the network from scratch requires large computational resources. Self-supervised learning focuses on mining its own supervised information from large-scale unsupervised data using auxiliary tasks, and training the network with this constructed supervised information so that it can learn representations that are valuable for downstream tasks. Such representations are usually generic [10], which not only improves the performance of the network, but also allows pre-training and preserving the model parameters to reduce the computation time. To this end, we first conducted heartbeat segmentation on QT Database (QTDB), MIT-BIH Arrhythmia Database (MITDB) and MIT-BIH Normal Sinus Rhythm Database (NSRDB). After preprocessing, three signal transformations were performed on the heartbeat signal, and the unlabeled original heartbeat and the three transformed heartbeats were fed into the base network together for self-supervised learning. The base network is optimized by Densenet [11] as the backbone, introducing attention module and feature pyramid module. Subsequently, the convolutional layer, which is contributed for high-level feature learning of ECG, was frozen and transferred to the downstream task network and further learned the labeled signals to complete the training of the localization network of ECG characteristic points.

## 2. Materials and Methods

### 2.1. Architecture

#### 2.1.1. Base Model

Our base network used a modified Densenet network to fully extract the features of ECG heartbeats using hopping layer connection structure. We incorporated convolutional block attention modules (CBAM) to discriminate and detect more subtle position features by combining spatial attention and channel attention mechanisms together. We also conducted feature scaling at different scales by introducing feature pyramid pooling (FPP) structure to improve detection accuracy.

#### 2.1.2. Self-Supervised Learning Structure

**Figure 1:**
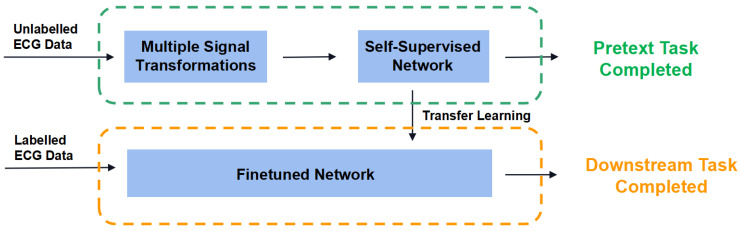
The framework of the proposed self−supervised network.

Our proposed self-supervised learning framework consisted of two learning phases. Step 1: Pretext task stage. Referring to [12], different signal transformations were applied to segmented heartbeats, respectively. Then we conducted the recognition task, which was called pretext task, discriminating the difference between the original and transformed signals. During this process, our model could fully learn the advanced features of ECG heartbeat signal. Then we froze the convolutional layer and saved the model parameters. Step2: Downstream task stage. The model parameters saved in the first stage were used to initialize the finetuned network. Then the data and labels from the QT dataset were fed into the finetuned network for learning and fine-tuning of the fully connected layer to finally complete the task of characteristic point localization. Figure 1 illustrates our network framework for self-supervised learning.

### 2.2. Datasets

#### 2.2.1. Data for Pretext Task

The MIT-BIH Arrhythmia Database(MITDB) contains 48 records of half-hour ambulatory ECG signals from 47 subjects, with each record annotated by two or more cardiologists for beat type. The recordings were digitized at 360 samples per second [13].

The MIT-BIH Normal Sinus Rhythm Database(NSRDB) includes 18 long-term ECG recordings of subjects. Subjects included in this database were found to have had no significant arrhythmias. The recordings were digitized at 128 samples per second [13].

There is no annotation for the position of characteristic points in both above-mentioned datasets. In our study, MITDB and NSRDB are suitable for self-supervised learning in the pretext task due to the presence of a large number of beats of the same type (type ‘N’) in it as in QT Database(QTDB) which has similar amplitude and waveform characteristics.

Since the pretext task did not require characteristic point labels, we only needed to cut out all heartbeats from QTDB in this stage. So did the heartbeats of type ‘N’ from MITDB and NSRDB. Then we resampled all of them to ensure a fixed signal length of 300. Referring to the segmentation approach of [14], we used the peak position of QRS wave as the reference point for heartbeat cutting. Finally, we obtained 79,643 heartbeats of type ‘N’ from MITDB and 309,384 heartbeats of type ‘N’ from NSRDB.

**Figure 2:**
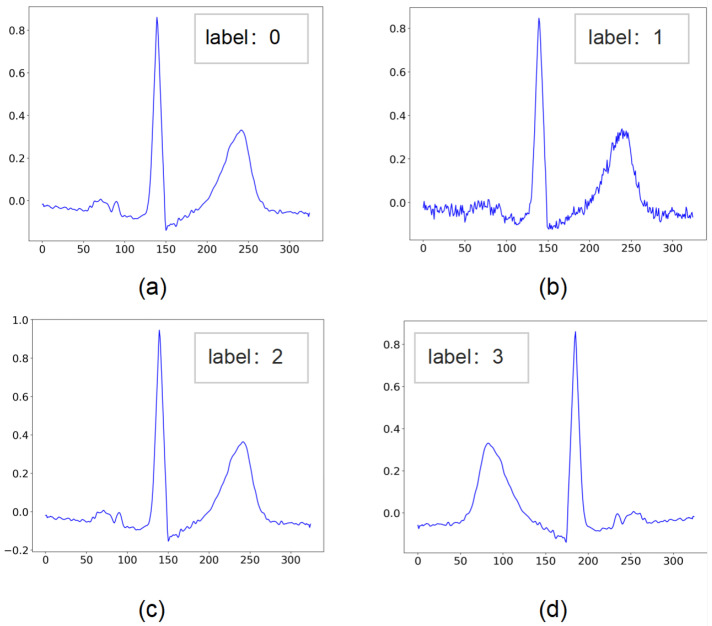
The original signal and the transformed signal, corresponding to the labels automatically generated by the transformation order. (**a**) Original signal, corresponding to label 0. (**b**) Noise Addition transformation, corresponding to label 1. (**c**) Scaling transformation, corresponding to label 2. (**d**) Temporal Inversion transformation, corresponding to label 3.

We performed three signal transformations including noise addition, scaling, and temporal inversion on the original heartbeats for data preparation of pretext task. After the transformations, four signals including the original signal are stacked to construct the input matrix X. We set the original signal as category 0, and the remaining three transformed signals correspond to categories 1∼3 in turn as labels Y for the multi-classification task. These category tags are so called ‘pseudo-labels’ for the whole self-supervised learning task. These labels are generated automatically in the order of transformations applied on the origin signal, without providing any manual annotations. Referring to [15] in which similar way was used to identify human activities, we fed tuples of inputs and pseudo-labels (X*i*, Y*i*) into the pretext network, where *i* denotes the *i*th transformation on the signal. By optimizing the loss function of this classification task, we can improve the ability of our self-supervised network to discriminate these four signals, during which the self-supervised network can fully learn the features of the original signals. Figure 2 shows signals before and after transformation and their automatically generated labels.

#### 2.2.2. Data for Downstream Task

**Figure 3:**
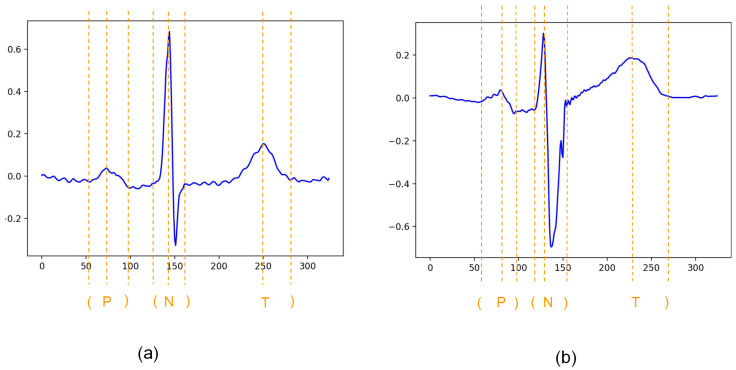
Annotations of each heart beat in the QT dataset. (**a**) Annotations of heartbeat 1, (**b**) Annotations of heartbeat 2.

Our study used QTDB for downstream tasks. QTDB consists of 105 2-lead recordings sampled at 250 Hz. It includes normal sinus rhythm, ischaemic and non-ischaemic ST segments, slow ST-segment drift, transient ST-segment depression and sudden cardiac death. At least 30 heartbeats in each record (3623 in total) were manually annotated by two experts. The specific annotations are shown in Figure 3, where “P” and “T” represent the peak of P and T waves, respectively, and “N” represents the normal heartbeat. The symbols “(” and “)” represent the onset and termination points of each wave, respectively, with the onset point of the T wave is not annotated. In our study, a large number of beats of the same type (type ‘N’) as in QTDB with similar amplitude and waveform characteristics are suitable for the self-supervised learning in the previous task due to the presence of a large number of beats of the same type (type ‘N’) in the MIT-BIH database. In our study, due to the presence of a large number of beats of the same type (type ‘N’) with QTDB in MITDB and NSRDB, which have similar amplitude and waveform characteristics, it’s suitable of the two datasets to be used for the self-supervised learning in the previous task.

We manually eliminated the records that did not contain all 8 characteristic points from the 105 records in QTDB, and ended up with 97 records. The above signals were passed through discrete wavelet transform (DWT) to reduce the noise of ECG signals. Referring to [16], we chose the sixth-order Daubechies wavelet function as the mother wavelet to decompose the ECG signal and to perform reconstruction. After denoising we conducted the segmentation of the heartbeats. We took 100 sampling points before the QRS peak points and 200 sampling points after the QRS peak points annotated by the experts, respectively. The time span of each heartbeat is 1.2 s (the sampling rate of QTDB is 250 Hz). The cut heartbeats were then upsampled to 325 sampling points to generate X, a 1D matrix of 1 × 325. At the same time, we processed the expert’s annotations into Y, a 1D matrix of 1 × 8. The tuples (X, Y) were then fed into the model as pairs for training.

### 2.3. Methodology

Our model consists of three main components: dense hop layer connection, convolutional block attention module (CBAM), and feature pyramid pooling module (FPP).

#### 2.3.1. Dense Hop-Layer Connection

Deeper deep learning networks imply better nonlinear representation and can fit more complex feature inputs. However, as the network deepens, it can lead to instability of the gradients along with gradient disappearance or gradient explosion. This situation leads to the degradation of the network performance, which is called degradation problem. It was ResNet that first introduced skip connection to solve the degradation problem. ResNet transmits the information from the initial layer to deeper layers by matrix addition. The main difference between DenseNet and ResNet is that DenseNet concats the output feature map of a layer with the next layer instead of summing up, which is so called feature reusability. The formula of Dense hop-layer connection is shown in Equation (1).

(1) $$X\left(t\right)=H\left(\left[X_{0},X_{1},X_{2},\ldots ,X_{i},\ldots ,X_{\left(t-1\right)}\right]\right)$$

which denotes the output X(t) of a *t*−layer network. $X_{i}$ is the output of the *i*th layer of the feed forward neural network; [$X_{0},X_{1},X_{2},\ldots ,X_{\left(t-1\right)}$] denotes the stitching of the output feature maps from layer 0 to layer *t* − 1, and *H*(·) denotes the non-linear transformation, including a combination of BN, ReLu, and convolutional layers.

Since the 1 × 1 convolutional kernel of each dense layer can reduce the number of input feature maps, DenseNet can learn the feature maps with fewer parameters than ResNet.

#### 2.3.2. Convolutional Block Attention Module

**Figure 4:**
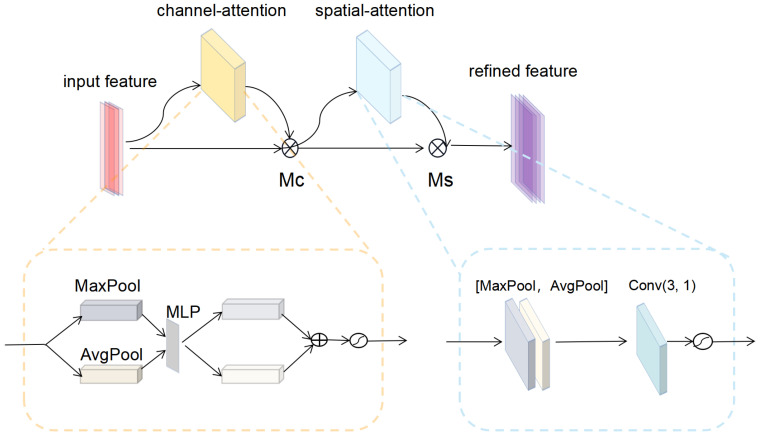
Convolutional block attention module (CBAM) structure. The input features are multiplied by the channel attention map output from the channel attention module. The multiplied features are then processed by the spatial attention module to obtain a spatial attention map. The spatial attention map is then multiplied by input features from the spatial attention module to obtain the refined features. Conv(3, 1) denotes a convolutional layer with a convolutional kernel size of 3 and an output channel number of 1. ‘+’ denotes the corresponding element summation and ’∫’ denotes the sigmoid function.

In this subsection, we will introduce the CBAM, which is an attention mechanism module combining spatial and channel attention. It was proposed by Woo et al. The specific structure of CBAM is shown in Figure 4.

The emphasis of the channel attention module is focusing on what is the key information of input features. Our study aimed to focus on the onset, peak and termination points of different wavelets in heartbeat signal. In this module, input features were first passed through average pooling layer and maximum pooling layer before being fed together into a multilayer perceptron (MLP) network. The output of MLP was then passed through the sigmoid function to obtain the final weights *M*${}_{c}$ of the channel attention. The expression of *M*${}_{c}$ is shown in Equation (2).

(2) $$\begin{array}{cc} M_{c}\left(X\right) & =\sigma \left(MLP\left(AvgPool\left(X\right)\right)+MLP\left(MaxPool\left(X\right)\right)\right)\\ \; & \;=\sigma \left(W_{1}\left(W_{0}\left(X_{avg}^{c}\right)\right)+W_{1}\left(W_{0}\left(X_{max}^{c}\right)\right)\right) \end{array}$$

where *X* denotes the feature map fed to the attention module, σ represents the sigmoid function. $W_{0}\in R^{C/r\times C}$,$W_{1}\in R^{C\times C/r}$; $X_{avg}^{C}$ and $X_{max}^{C}$ denote the channel context descriptors generated by average pooling and maximum pooling, respectively. *C* is the number of channels.

The spatial attention module, on the other hand, focuses on the location of key information. Our study aimed to focus on the temporal characteristics of ECG wavelets, which means that the order of characteristic points of different wavelets could be correctly discriminated, suppressing possible errors due to the close position of adjacent points. In this module, input features were sequentially passed through average pooling layer and maximum pooling layer, and then the results were concatenated together as the input to the convolution layer. The expression of the weights *M*${}_{s}$ of spatial attention is:

(3) $$\begin{array}{cc} M_{s}\left(X\right) & =\sigma \left(f^{3\times 3}\left(\left[AvgPool\left(X\right);MaxPool\left(X\right)\right]\right)\right)\\ \; & =\sigma \left(f^{3\times 3}\left(\left[X_{avg}^{s};X_{max}^{s}\right]\right)\right) \end{array}$$

where *X* denotes the feature map fed to the attention module, σ represents the sigmoid function, $X_{avg}^{S}$ and $X_{max}^{S}$ denote the spatial context descriptors generated by average pooling and maximum pooling, respectively. 3×3 denotes the size of the convolution kernel.

#### 2.3.3. Feature Pyramid Pooling Module

**Figure 5:**
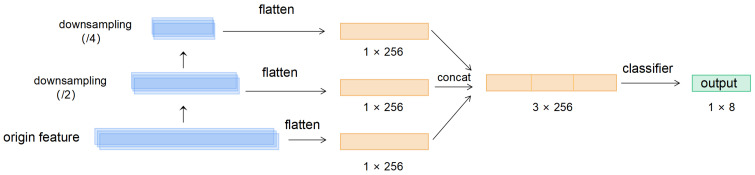
Feature Pyramid Structure (FPN).

After conducting multiple convolutional and pooling operations on the input ECG signal, we introduced a feature pyramid module for heartbeat signal in order to extract more information from feature map at multiple scales, as shown in Figure 5. We downsampled the feature maps generated by the intermediate layer twice and passed them uniformly through a fully connected layer (1 × 256) with the same output dimension. After concatenating the results of the multiscale deflation with the original features and sending it to the fully connected layer, we accomplished the prediction of the characteristic point detection results.

**Figure 6:**
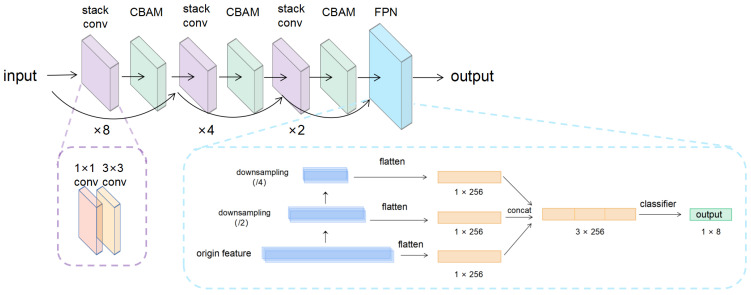
The proposed detection network.

Finally, we constructed the detection network as shown in Figure 6. After the heartbeats were preprocessed, they were fed into the repetitive stacked convolution layer and Dense Block module with CBAM module introduced into the middle of each repetitive unit. After the features were fully extracted by the hopping layer structure and attention mechanism, the generated intermediate features were fed into the FPP module for multi-scale feature extraction, and then the final fully connected layer predicted the positions of the eight characteristic points. Table 1 details the structure and relative parameters of our detection network.

### 2.4. Experiments

#### 2.4.1. Pretext Task

**Figure 7:**
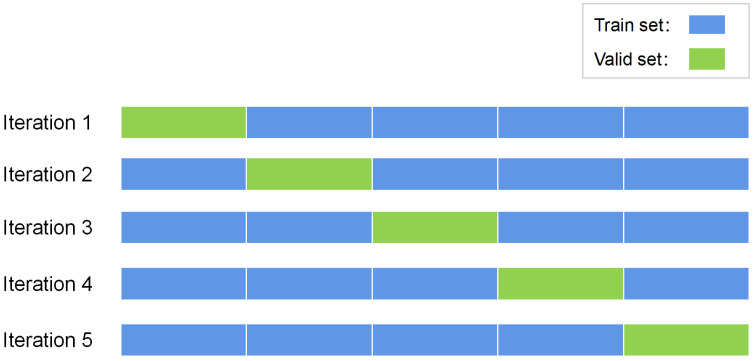
Division of 5-fold cross-validation data.

We conducted a five-fold cross-validation on MITDB and NSRDB separately when training the datasets of pretext task. First, we divided the dataset into five subsets of data with equal amount of data. In each iteration, one of these subsets was sequentially selected as the validation set during training, and our pretext task model was trained on the remaining four subsets. The final accuracy of our model was obtained by averaging the accuracy of each iteration. The specific division method of the five-fold cross-validation is shown in Figure 7. We used cross-entropy as the loss function and chose Adam optimizer for the training of our pretext task model.

#### 2.4.2. Downstream Task

We set the ratio of training set and testing set for downstream task to be 8:2 and used the 5-fold cross-validation method to train the proposed model. Referring to [15], we used He initialization technique to initialize the network parameters. We used stochastic gradient descent with a fixed learning rate of 0.001 and a momentum parameter of 0.9.

We used L1 loss (i.e., mean absolute error (MAE)), calculating the mean deviation between the predicted position of each characteristic point and experts’ annotations as the loss function for the downstream task. Since the experts had 2 ECG leads available during annotation and the input of our network is one-lead, both leads were processed independently. The one with the smaller mean deviation was selected when processing the results. For each ECG recording, we used the mean deviation and standard deviation (m ± sd) as measure for all samples. We also used early stopping method to prevent over-training of our proposed model.

### 2.5. Experimental Environment

Operating system: Ubuntu; processor: NVIDIA GeForce RTX 3090; memory size: 24 G; programming platform: Pycharm, Python version 3.8.

## 3. Results

**Figure 8:**
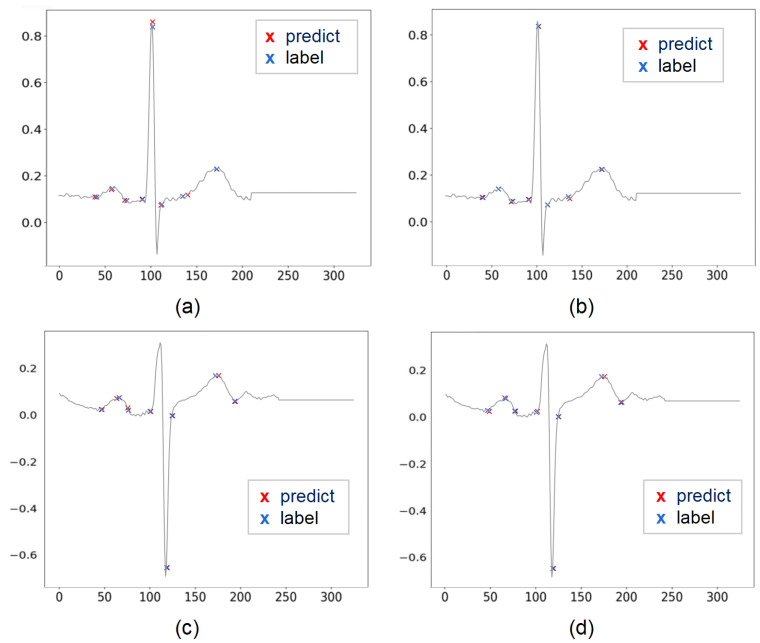
Predicted positions of fully supervised network and self-supervised network on the same heartbeat. (**a**) Predicted positions of fully supervised network on heartbeat 1. (**b**) predicted positions of self-supervised network on heartbeat 1. (**c**) Predicted positions of fully supervised network on heartbeat 2. (**d**) Predicted positions of self-supervised network on heartbeat 2.

The results of our study are shown in Table 2. Referring to [17], since the peak position of QRS complex wave was the segmentation criterion of the input heartbeats in our study, the QRS peak points were not taken into account in the result statistics. Figure 8 shows the regression results of the fully supervised network and the self-supervised network for different morphologies of some heartbeat characteristic points. Figure 8a,c both show the predicted positions of the fully supervised network proposed in our previous study [18], and Figure 8b,d corresponds to the positions predicted by our self-supervised network for the same heartbeat.

We can see from the comparison that the self-supervised network predicts the position of characteristic points more accurately than the fully supervised network. The mean value of the deviation of the position prediction of characteristic points does not exceed two samples and the standard deviation does not exceed six samples for both the fully supervised network and the self-supervised network.

## 4. Discussion

### 4.1. Comparative Analysis of Fully Supervised Network and Self-Supervised network

**Figure 9:**
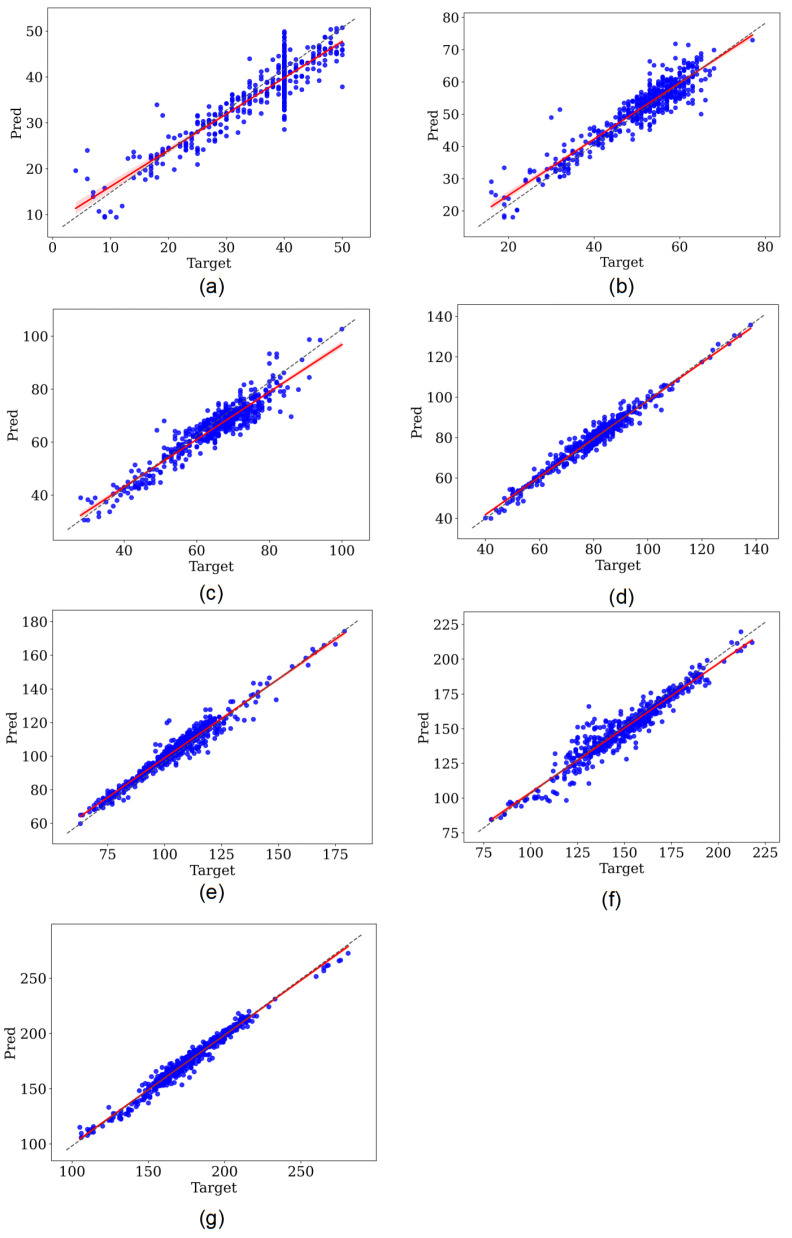
Regression plot of fully supervised network. (**a**–**g**) Correspond to the onset, peak, and termination points of P-waves, the onset and termination points of QRS waves, and the peak and termination points of T-waves, respectively.

**Figure 10:**
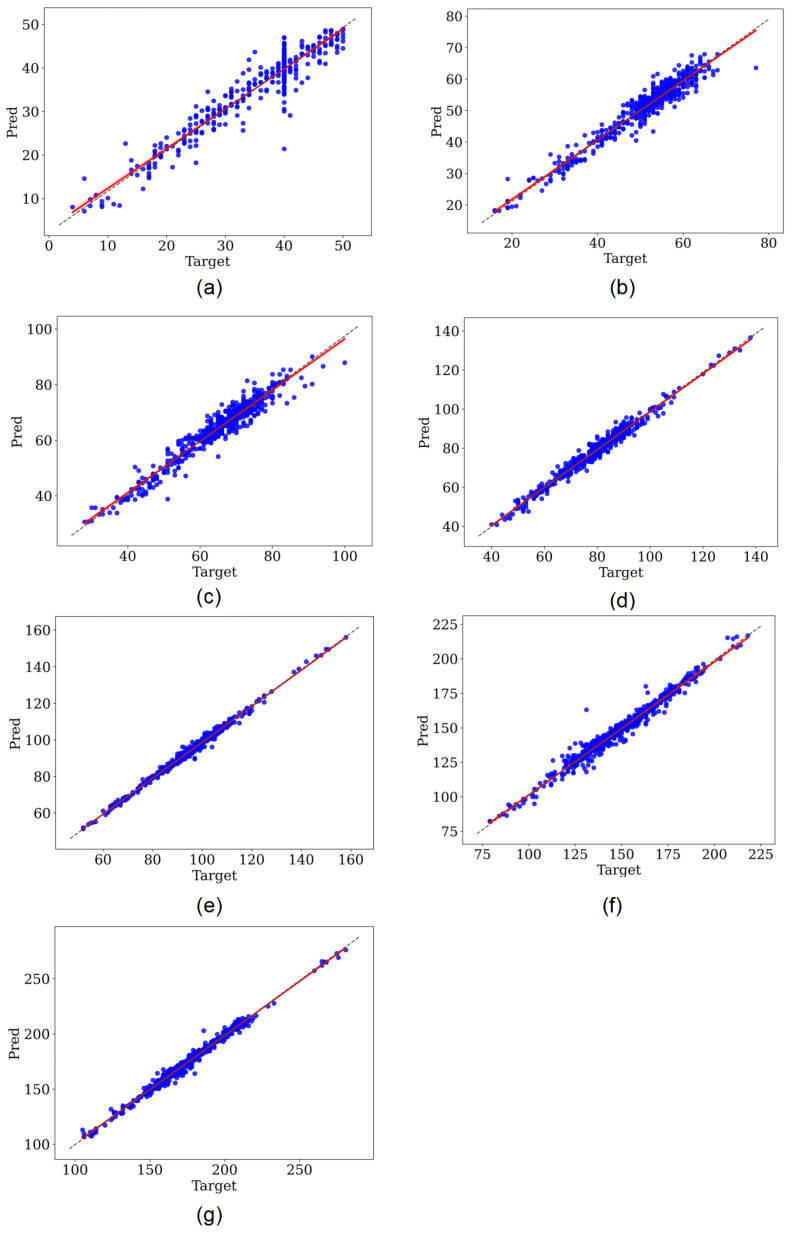
Regression plot of self-supervised network. (**a**–**g**) Correspond to the onset, peak, and termination points of P-waves, the onset and termination points of QRS waves, and the peak and termination points of T-waves, respectively.

**Figure 11:**
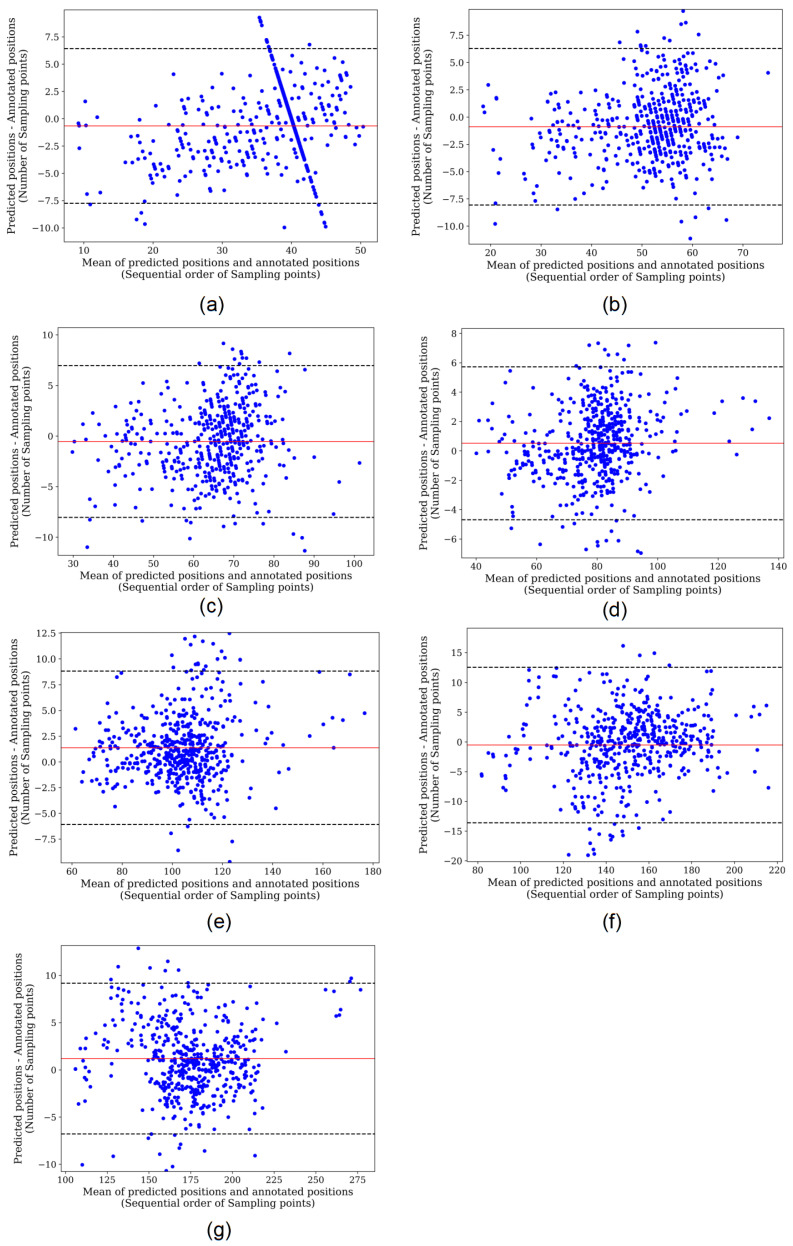
Bland–Altman plot of fully supervised network. (**a**–**g**) Correspond to the onset, peak, and termination points of P-waves, the onset and termination points of QRS waves, and the peak and termination points of T-waves, respectively.

**Figure 12:**
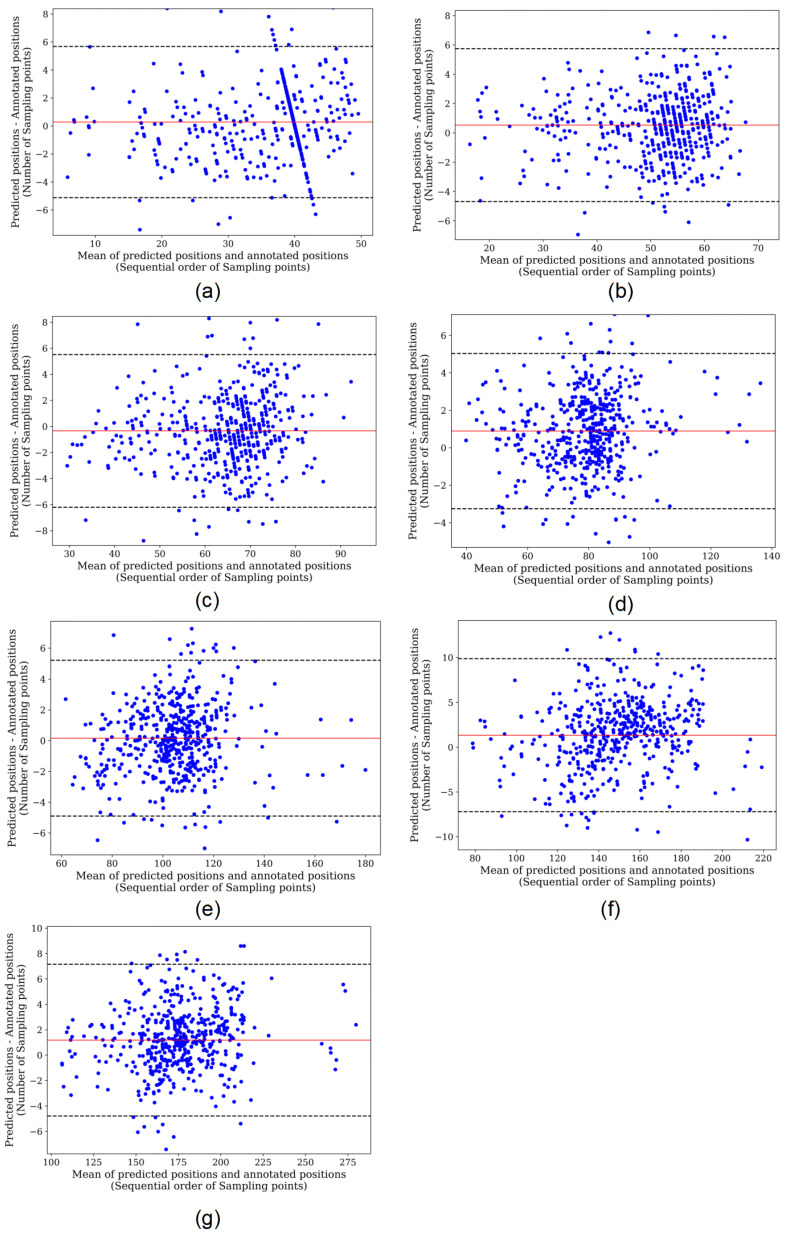
Bland–Altman plot of self-supervised network. (**a**–**g**) Correspond to the onset, peak, and termination points of P-waves, the onset and termination points of QRS waves, and the peak and termination points of T-waves, respectively.

For a more explicit comparison, we calculated the absolute deviations of the models fully supervised trained and self-supervised trained based on the QT dataset, as shown in Table 3. It can be seen that the self-supervised model has better performance compared to the fully supervised model when trained on the same dataset. We used regression analysis and Bland–Altman plots to visualize the detection results of the two models. The regression plots allow to observe the approximation of the predicted positions and the annotated positions with the help of trend lines. When the trend line fits closely to the straight-line y=x, it means the model performs well in regression detection. Figure 9 and Figure 10 represent the regression results of the positions of the seven characteristic points predicted by the fully supervised model and the self-supervised model, respectively, where the *x*-axis represents the positions of the annotated characteristic points and the *y*-axis represents the predicted positions of the model. We can see that the self-supervised model fits more closely to the straight line y=x for each characteristic point, while the fully supervised model fits significantly worse at the whole P-wave and T-wave peak points. Bland–Altman plots were used to evaluate the correlations between the measured values. With Bland–Altman plots, we can see the 95% limit of agreement (LOA) of the data, of which smaller value indicates better performance of the model [19]. We used the positions annotated by the experts as the criterion comparing with those of the fully supervised and self-supervised models, respectively. Figure 11 and Figure 12 represent the Bland–Altman plots of the positions of the seven characteristic points predicted by the fully supervised network and the self-supervised network, respectively, where the *x*-axis represents the mean sequence order of predicted positions and annotated positions, and the *y*-axis represents the difference of predicted positions and annotated positions. It can be seen that the confidence intervals of the Bland–Altman plots of the self-supervised model are smaller, except for the less obvious result of the termination point of the P-wave, which means that the standard deviation of the predicted positions of the self-supervised model is smaller.

### 4.2. Comparative Analysis of Self-Supervised Network Pretrained on Different Database

As can be seen in Table 2, the results of the characteristic point localization based on the MITDB and NSRDB have less bias, but more variance compared to those on QTDB, which is unavoidable when using different datasets for self-supervised pre-training. This is due to the MITDB and NSRDB has a larger amount of same type heartbeats, and thus more heartbeat features can be extracted. So the mean of deviation is small. In contrast, QTDB has a small fraction of abnormal heartbeats compared to those selected from the MITDB and NSRDB, which ultimately leads to a larger variance of deviation for self-supervised learning using MITDB and NSRDB. In the meanwhile, the model pre-trained on NSRDB predicted more accurately than the model pre-trained on MITDB, with smaller standard deviations of the predicted positions except for that of the T-wave peak. This may owe to the fact that NSRDB has a larger amount of data compared to MITDB, which allowed the model to learn features adequately.

### 4.3. Comparative Analysis with Other Heartbeat Characteristic Points Detection Results

In order to compare the performance of our method and other methods, we list several traditional and deep learning methods in Table 4. In order to make the role of each module in this study stand out, we implemented a baseline network simple-dense, which used the same number of dense blocks and transition layers as our model without the CBAM and FPP modules. We calculated the mean absolute error of mean values of all points for each method, as shown in Table 4. It can be seen that the self-supervised model proposed in this manuscript has a significant performance improvement from the perspective of mean deviation, especially for the detection of p-wave onset, peak and termination points. However, the self-supervised model has poorer predictions at the T-peak and T-off positions compared to our previously published fully supervised network. We speculate that this may be due to the fact that QTDB contains some ST-segment drifting, transient ST-segment depression heartbeats [20], and the self-supervised model was trained with normal heartbeats in both MITDB and NSRDB in pretext task, resulting in a bias in the detection of the downstream task. According to our mean absolute error of mean deviation calculated in Table 4, our self-supervised model has smaller mean and standard deviation compared to MP-EKF [21], which used the traditional algorithm. Compared with the deep learning model U-Net [8], our detection deviation in P-wave and T-wave are smaller. The above shows that our proposed model has significant advantages in detecting ECG characteristic points, especially in P-wave.

### 4.4. Analysis of the Validity of the Model Construction

**Figure 13:**
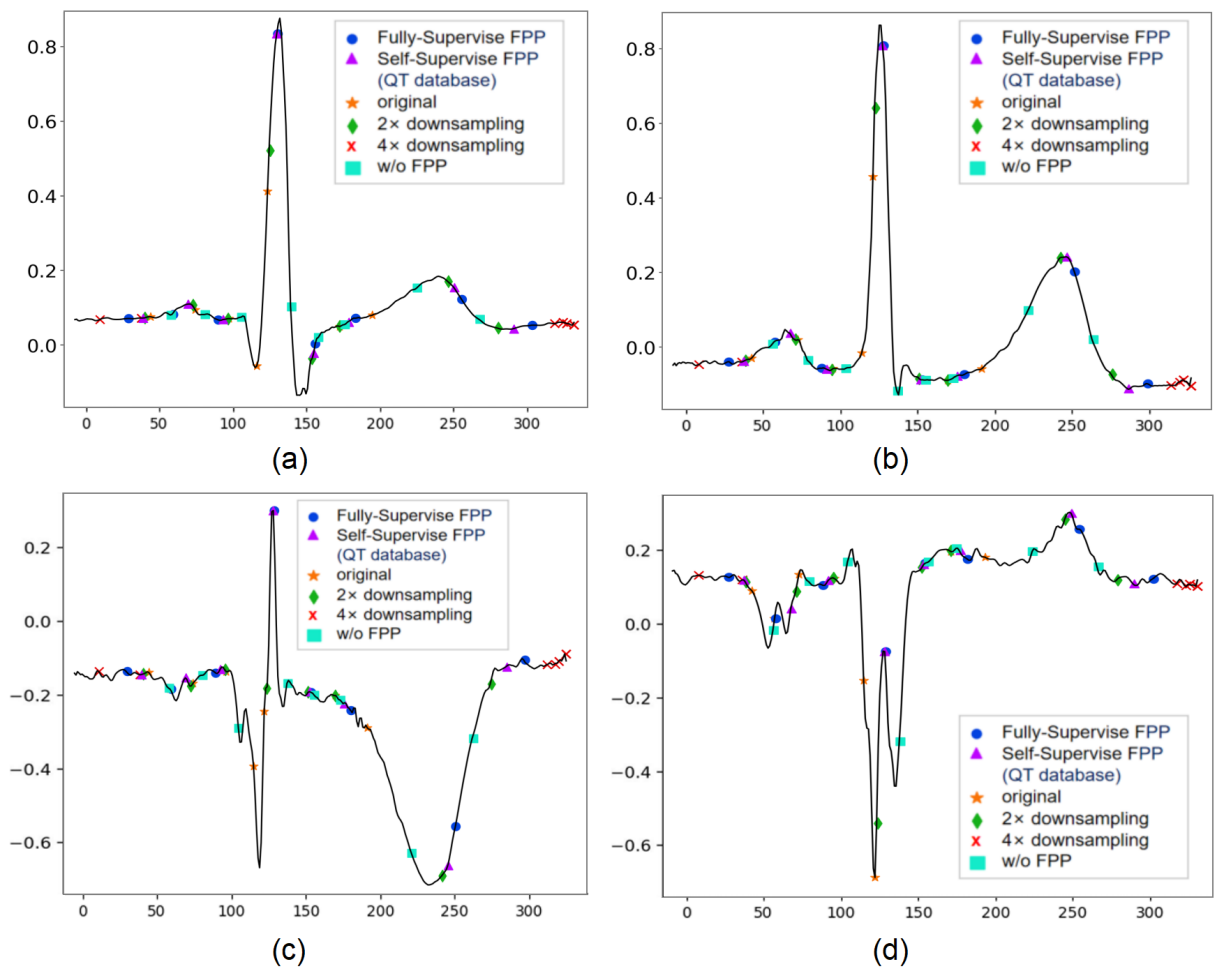
Position of characteristic points corresponding to feature maps at different scales, where the blue solid circle and purple upper triangle denote the predicted positions of fully supervised network and self-supervised network, respectively. Furthermore, the orange star, green rhombus and red fork sign indicate the original feature map, the predicted positions obtained by the feature maps downsampled by a factor of two and four, respectively. The predicted positions of fully supervised network without FPP module are marked with aqua blue square. (**a**) Regression results for heartbeat 1, (**b**) regression results for heartbeat 2, (**c**) Regression results for heartbeat 3 and (**d**) regression results for heartbeat 4.

**Figure 14:**
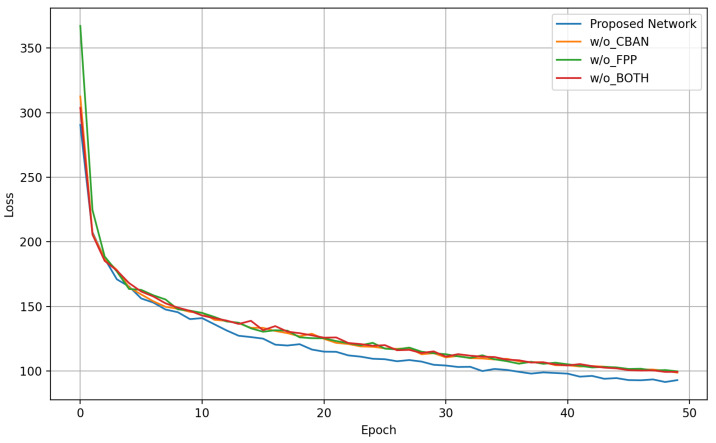
Loss curve of comparison experiment.

To further validate the effectiveness of the main modules of the model in this manuscript, we conducted control experiments for each module introduced in our work. The experimental results are shown in Table 5. We defined our previously published fully supervised-based network as model 1, the self-supervised model pre-trained on QTDB as model 2, the model with the CBAM module removed in model 2 as model 3, the model with the feature pyramid module removed in model 2 as model 4, and the model with both the CBAM module and the feature pyramid module removed in model 2 as model 5. By comparing model 3 with model 2, it was found that the model performance decreases significantly without the CBAM module, and the mean absolute error of mean deviation expands to 4.52 ms. We speculate that this is due to the lack of channel attention module to focus on the important parts of the input, such as the amplitude and morphology of each wavelet and the lack of spatial attention to focus on the order of characteristic points. It is apparent that both the mean and standard deviation are substantially larger when comparing model 2 and model 4, which indicates a decrease in model performance. We speculate that without the feature pyramid pooling module, the model cannot use the relative positions of the waves and the spacing scales of the different waves for feature point identification. Figure 13 shows the positions of the characteristic points corresponding to the feature maps at different scales of FPP structure of multiple morphological heartbeats. It can be seen that both the self-supervised network structure and the fully supervised network structure with FPP have better detection results. Model 5 performed poorly in both mean and standard deviation, which confirmed the performance improvement of our proposed module. Figure 14 shows the loss curve of the comparison experiment.

### 4.5. Limitations of Our Research

Our study also needs further exploration under the current constraints. On the one hand, since the public dataset for characteristic point detections is not large enough, both the number of patients and the types of ECG heartbeats need to be expanded more broadly. On the other hand, methods to improve the accuracy of cutting complete heartbeats are also the focus of our future research. Since the results of the self-supervised pretext task vary widely on different datasets, this also presents an issue that we hope to improve in the future, and which requires us to propose some strategies for personalized features in our future work.

## 5. Conclusions

In this manuscript, we propose a new deep-learning-based method with self-supervised learning for detecting the characteristic points of ECG beats. We selected QTDB, MITDB and NSRDB as the downstream task and pretext task database, respectively. After preprocessing and different morphological feature transformations, unlabeled heartbeat signals were fed into the base network for self-supervised learning. We saved the convolutional parameters to initialize the network for the downstream tasks and successfully completed the task of characteristic point localization of the ECG signals. DenseNet is employed as the base network, the CBAM module is added to enhance the extraction of valid information, and the feature map is multi-scaling deflated to improve information extraction. The experimental results show that the mean error of the characteristic point detections is less than one sample point (except the QRS-peak) and the standard deviation is less than five sample points (except for the QRS-peak). The obtained results in our manuscript suggest that compared with the fully supervised model, our proposed deep learning model based on self-supervised learning has smaller detection deviation. The results of this study provide a basis for ECG-information extraction based on characteristic points of the heart beat.

## Figures and Tables

**Table 1:** Details of the Detection Network Structure.

Type	Input Shapes	Output Shapes
Input	(324, 1)	(324, 1)
Conv	(324, 1)	(324, 64)
Pooling	(324, 64)	(162, 64)
Dense Block	(162, 64)	(162, 320)
TransitionLayer	(162, 320)	(81, 160)
Dense Block	(81, 160)	(81, 288)
TransitionLayer	(81, 288)	(40, 144)
Dense Block	(40, 144)	(40, 208)
TransitionLayer	(40, 208)	(20, 104)
Pooling	(20, 104)	(21, 104)
Conv	(21, 104)	(20, 128)
Dropout	(20, 128)	(20, 128)
Flatten	(20, 128)	2560
Fully-connected	2560	256
Pooling (downsampling 2×)	(20, 128)	(10, 128)
Flatten	(10, 128)	1280
Fully-connected	1280	256
Pooling (downsampling 4×)	(10, 128)	(5, 128)
Flatten	(5, 128)	640
Fully-connected	640	256
Concatenation	256 × 3	768
Fully-connected	768	256
Fully-connected	256	8

**Table 2:** Results of Our Model on Public Database.

Method	Pretext Task Database	P-On m ± s (ms)	P-Peak m ± s (ms)	P-Off m ± s (ms)	QRS-On m ± s (ms)	QRS-Off m ± s (ms)	T-Peak m ± s (ms)	T-Off m ± s (ms)
Fully Supervised [18]	/	−0.32 ± 18.08	−0.56 ± 17.6	−5.96 ± 16.84	−5.8 ± 14.12	−6.24 ± 18.76	**−0.2 ± 31.36**	0.84 ± 27.24
Self-Supervised	MITDB	0.12 ± 20.96	0.26 ± 16.16	−0.8 ± 15.28	2.36 ± 9.36	−2.72 ± 19.2	−0.8 ± 20.56	−2.8 ± 23.28
Self-Supervised	NSRDB	**−0.08 ± 11.56**	**−0.04 ± 11.24**	0.92 ± 12.36	**−2.2 ± 8.32**	**0.48 ± 9.16**	−2.36 ± 27.24	**−0.68 ± 21.64**
Self-Supervised	QTDB	−0.24 ± 10.04	−0.48 ± 11.69	**−0.28 ± 10.19**	−3.72 ± 8.18	−4.12 ± 13.54	−0.68 ± 20.42	1.34 ± 21.04

**Table 3:** Comparison of models on mean absolute deviation.

Method	Pretext Task Database	P-On m ± s (ms)	P-Peak m ± s (ms)	P-Off m ± s (ms)	QRS-On m ± s (ms)	QRS-Off m ± s (ms)	T-Peak m ± s (ms)	T-Off m ± s (ms)
Fully Supervised [18]	/	8.52 ± 8.48	8.32 ± 8.22	10.16 ± 8.61	7.35 ± 5.94	8.09 ± 7.29	12.76 ± 12.92	8.45 ± 8.74
Self-Supervised	QTDB	7.75 ± 8.18	7.52 ± 7.01	8.89 ± 7.88	6.63 ± 5.42	7.19 ± 6.6	11.47 ± 11.32	8.26 ± 8.53

**Table 4:** Comparative Analysis of Results with Other Heartbeat Characteristic Points Detection.

Method	Database	P-On m ± s (ms)	P-Peak m ± s (ms)	P-Off m ± s (ms)	QRS-On m ± s (ms)	QRS-Off m ± s (ms)	T-Peak m ± s (ms)	T-Off m ± s (ms)	MAE of Mean Deviation (ms)
Fully Supervised [18]	QTDB	−0.32 ± 18.08	−0.56 ± 17.6	−5.96 ± 16.84	−5.8 ± 14.12	−6.24 ± 18.76	**−0.2 ± 31.36**	0.84 ± 27.24	2.84
Self-Supervised	QTDB	**−0.24 ± 10.04**	**−0.48 ± 11.69**	**−0.28 ± 10.19**	−3.72 ± 8.18	−4.12 ± 13.54	−0.68 ± 20.42	1.34 ± 21.04	**1.55**
Simple-Dense (baseline)	QTDB	2.2 ± 18.16	4.6 ± 18.08	−2.04 ± 13.11	3.72 ± 15.24	−8.78 ± 18.32	−1.12 ± 30.27	1.68 ± 22.2	3.45
TWA	QTDB	N/A	N/A	N/A	2.8 ± 7.7	2.7 ± 9.7	−2.6 ± 12.2	−2.7 ± 20.7	2.7
MsPE	QTDB	0.5 ± 15.1	5.1 ± 10.9	0.5 ± 15.0	0.9 ± 8.5	**−0.4 ± 9.6**	−4.5 ± 14.7	**0.6 ± 20.3**	1.79
MP-EKF	QTDB	16 ± 37	5 ± 34	−10 ± 34	NA	NA	−3 ± 24	−16 ± 35	10.0
U-Net	QTDB	1.54 ± 22.89	N/A	0.32 ± 4.01	**−0.07 ± 8.37**	3.64 ± 12.55	N/A	4.55 ± 31.11	2.02

**Table 5:** Results of the Control Experiment.

Method	P-On m ± s (ms)	P-Peak m ± s (ms)	P-Off m ± s (ms)	QRS-On m ± s (ms)	QRS-Off m ± s (ms)	T-Peak m ± s (ms)	T-Off m ± s (ms)	MAE of Mean Deviation
model 1 ^1^	−0.32 ± 18.08	−0.56 ± 17.6	−5.96 ± 16.84	−5.8 ± 14.12	−6.24 ± 18.76	**−0.2 ± 31.36**	**0.84 ± 27.24**	2.84
model 2	**−0.24 ± 10.04**	**−0.48 ± 11.69**	**−0.28 ± 10.19**	**−3.72 ± 8.18**	**−4.12 ± 13.54**	−0.68 ± 20.42	1.34 ± 21.04	**1.17**
model 3	2.12 ± 13.36	6.24 ± 13.72	4.92 ± 15.04	5.24 ± 11.72	−6.4 ± 14.52	1.16 ± 24.36	−4.92 ± 27.8	4.43
model 4	−1.76 ± 12.36	−2.60 ± 11.4	−4.16 ± 12.92	−4.36 ± 9.28	−6.27 ± 14.0	−2.2 ± 29.64	−1.52 ± 23.6	3.27
model 5	−2.32 ± 14.24	−4.36 ± 13.24	−6.08 ± 14.46	−7.4 ± 10.52	−7.24 ± 16.6	8.84 ± 26.28	3.52 ± 24.76	5.68

^1^ model 1: Fully Supervised Model [18]; model 2: Self-Supervised Model trained on QTdb; model 3: Self-Supervised w/o CBAM; model 4: Self-Supervised w/o Feature-pyramid; model 5: Self-Supervised w/o Both CBAM and Feature-pyramid.

## Data Availability

The dataset is available at https://www.physionet.org/about/database/.

## Abbreviations

The following abbreviations are used in this manuscript:

ECG	Electrocardiogram
QTDB	QT Database
MITDB	MIT-BIH Arrhythmia Database
NSRDB	MIT-BIH Normal Sinus Rhythm Database
CBAM	Convolutional Block Attention Module
MAE	Mean Absolute Error
